# Acute Endocrine and Lipid Responses to Extreme Ultramarathon Running Across 100, 308, and 622 km Events

**DOI:** 10.3390/life16091450

**Published:** 2026-08-31

**Authors:** Kyung-A Shin, Shunzhe Piao, Young-Joo Kim

**Affiliations:** 1 Department of Clinical Laboratory Science, Shinsung University, Chungnam 31801, Republic of Korea; mobitz2@hanmail.net; 2 School of Social Sports, Shenyang Sport University, Shenyang 110102, China; 2428595988@qq.com; 3 School of Sports Science, Sungshin Women’s University, Seoul 02844, Republic of Korea

**Keywords:** ultramarathon, extreme endurance exercise, endocrine response, lipid metabolism, gonadotropins, thyroid hormones, C-peptide, metabolic responses

## Abstract

Extreme ultramarathon running provides a unique human model for examining physiological responses under sustained metabolic stress. This study compared acute endocrine and lipid responses across 100, 308, and 622 km ultramarathon events, with particular attention to whether these responses were uniformly amplified with increasing race distance or differed according to event characteristics. Forty-four experienced male ultramarathon runners participated in the study (100 km group: *n* = 17; 308 km group: *n* = 18; 622 km group: *n* = 9). Venous blood samples were collected within 2 h before race start and generally within 5 min after race completion, and post-race biochemical concentrations were corrected for participant-specific plasma volume changes. Insulin and triglycerides decreased significantly across all three events, whereas T3 and HDL-C increased across all events. FSH and C-peptide decreased significantly in the 100 and 308 km groups, and FT4 increased significantly in these two groups. LH showed a significant time × event group interaction, with a significant reduction in the 100 km group and no significant within-group change in the 308 or 622 km groups. Because LH secretion is pulsatile and only one venous sample was obtained at each pre- and post-race time point, these findings should be interpreted as acute differences in circulating LH concentrations rather than evidence of altered gonadotropin regulation or hypothalamic–pituitary–gonadal axis suppression. Total cholesterol decreased significantly only after the 622 km event, whereas LDL-C decreased after the 308 and 622 km events. The observed endocrine and lipid response patterns differed across ultramarathon events characterized by distinct cumulative durations, pacing characteristics, nutritional exposure, hydration conditions, and recovery opportunities. Extreme ultramarathon running may therefore serve as a useful physiological model for investigating context-dependent endocrine and lipid responses to prolonged endurance stress.

## 1. Introduction

Extreme ultramarathon running represents a rare human model for examining acute endocrine and metabolic responses under prolonged physiological stress. Recent evidence suggests that during sustained physical activity, total energy expenditure does not increase indefinitely but approaches an upper metabolic ceiling of approximately 2.5 times basal metabolic rate as exercise duration becomes prolonged [1]. Ultramarathon events, particularly those extending beyond conventional single-day endurance races, place the human body close to this physiological boundary and impose substantial demands on energy homeostasis, endocrine regulation, and substrate metabolism [2,3]. However, the biological responses to extreme ultramarathon running may not simply become larger as race distance increases. Instead, they may differ across events characterized by differences in cumulative duration, pacing patterns, nutritional exposure, hydration status, sleep deprivation, environmental conditions, and intermittent recovery opportunities.

Endocrine responses are central to the regulation of energy availability during prolonged endurance stress [4]. Luteinizing hormone (LH) and follicle-stimulating hormone (FSH), as components of the hypothalamic–pituitary–gonadal axis, regulate male reproductive endocrine function [5]. Prolonged endurance exercise and severe physiological stress may alter gonadotropin regulation and can induce transient changes in reproductive axis activity [6,7]. Thyroid hormones also play a key role in metabolic regulation by influencing basal metabolic rate, mitochondrial function, oxygen consumption, and lipid oxidation during prolonged exertion [8,9]. In addition, insulin and C-peptide provide important information regarding pancreatic β-cell secretion and substrate availability because sympathetic activation and increased muscular glucose demand during endurance exercise can suppress insulin secretion while skeletal muscle maintains glucose uptake through insulin-independent mechanisms [10,11]. Therefore, simultaneous assessment of gonadotropins, thyroid hormones, and insulin-related markers may help characterize endocrine responses that support prolonged energy demand during extreme endurance exercise.

Lipid metabolism is another major component of physiological responses during ultra-endurance exercise. Acute and chronic endurance exercise can modify circulating lipid profiles, including reductions in triglycerides (TGs) and low-density lipoprotein cholesterol (LDL-C), as well as increases in high-density lipoprotein cholesterol (HDL-C) [12,13]. During ultramarathon running, the prolonged demand for adenosine triphosphate production increases reliance on fatty acid mobilization and oxidation, particularly as carbohydrate availability becomes limited [14]. Previous studies have reported acute changes in total cholesterol (TC), TGs, and lipoprotein fractions after ultra-endurance events [15]. However, lipid responses after extreme ultramarathon running may reflect a complex interaction among substrate utilization, hepatic lipoprotein handling, inflammatory signaling, nutritional intake, and recovery status rather than a simple linear effect of race distance.

Despite growing interest in the physiology of ultramarathon running, most previous studies have investigated single-distance events or have focused on isolated biomarker domains. Evidence indicates that muscle damage, inflammatory cytokines, endocrine hormones, and metabolic markers can change substantially after ultra-endurance races [2,16,17,18]. Direct comparisons of acute endocrine and lipid responses across markedly different ultramarathon events remain limited, particularly when the events differ in distance, duration, pacing structure, nutritional exposure, and opportunities for rest. This distinction is important because 100, 308, and 622 km ultramarathon events represent different physiological contexts: a shorter-duration event, an ultra-long continuous event, and an extreme multi-day endurance exposure. Comparing these events may help determine whether endocrine and lipid responses are uniformly amplified with increasing distance or whether they shift in pattern according to the broader event context and cumulative physiological load.

To address this gap, this study compared pre- and post-race levels in endocrine hormones, insulin-related markers, and lipid profiles among experienced male runners who completed 100, 308, or 622 km ultramarathon events. We framed these races as distinct extreme endurance conditions rather than as a simple distance gradient. We hypothesized that lipid responses would become more pronounced under longer cumulative exercise exposure, whereas endocrine responses would differ across event contexts rather than changing uniformly with race distance.

## 2. Materials and Methods

### 2.1. Participants and Study Protocol

**Figure 1:**
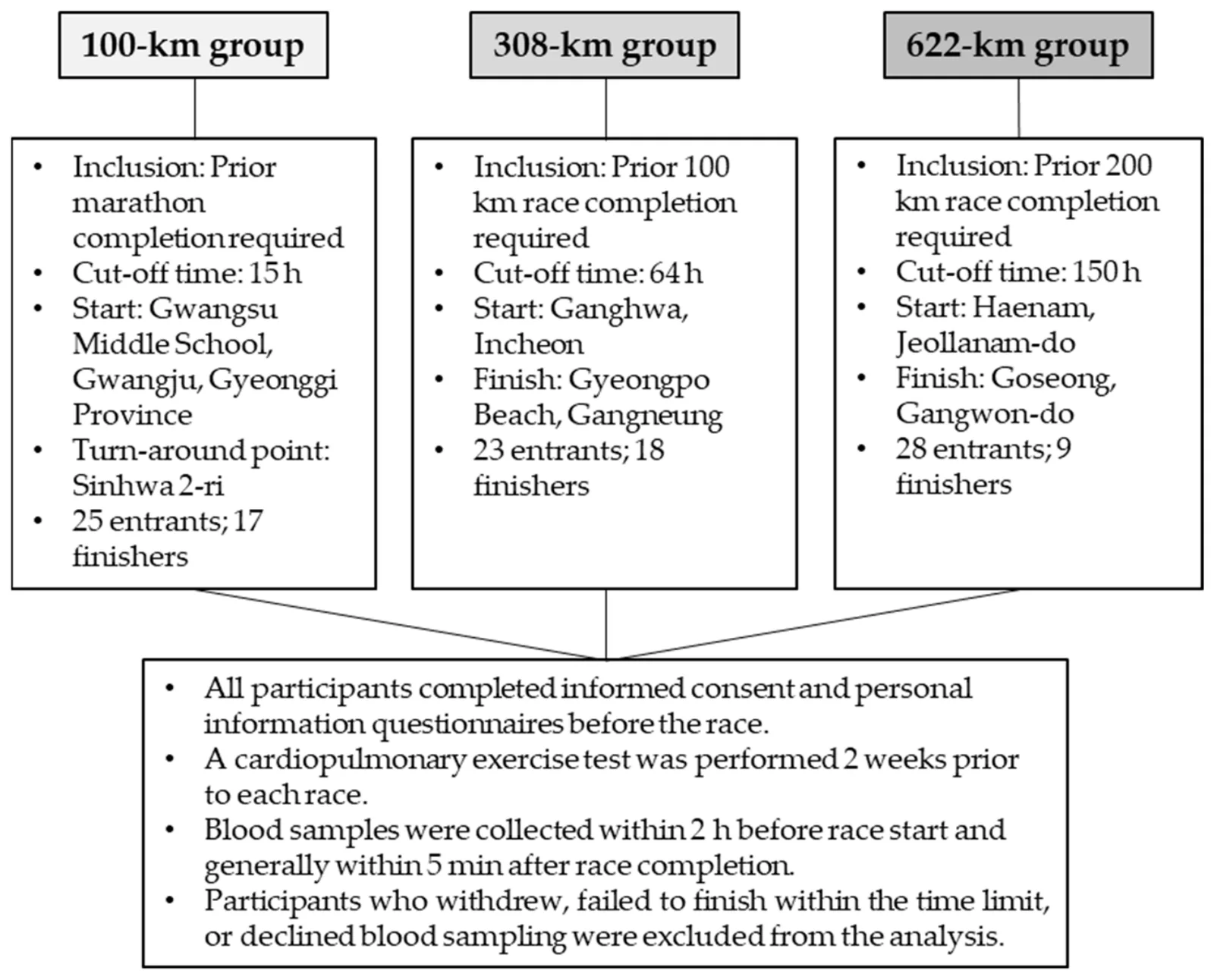
Race characteristics and study procedures.

Participant eligibility and study procedures are summarized in Figure 1. A total of 76 experienced ultramarathon runners volunteered for the study. This observational study was designed to compare pre- and post-race changes in endocrine hormones, insulin-related markers, and lipid profiles across three real-world ultramarathon events: 100, 308, and 622 km events. These events were treated as distinct extreme endurance conditions rather than as a simple linear distance gradient. Before participation, all runners received a full explanation of the study procedures and provided written informed consent. They also completed standardized questionnaires on anthropometric characteristics, medical history, and training background.

For the 100 km event, eligibility was restricted to runners who had completed at least one full marathon. The course started at Gwangsu Middle School in Gwangju, Gyeonggi-do, and turned back at Shinhwa 2-ri, with a cut-off time of 15 h. At the 50 km checkpoint (CP), split times were verified and carbohydrate-based meals and beverages were provided. Of the 25 runners who initially applied, 17 ultimately completed both the pre- and post-race blood sampling procedures and were included in the final analysis. At the start of the race, ambient temperature and relative humidity were 23 °C and 50%, respectively.

For the 308 km event, eligibility was restricted to runners who had completed at least one 100 km ultramarathon within the qualifying time. The course started in Ganghwa, Incheon, and ended at Gyeongpo Beach, Gangneung, with a cut-off time of 64 h. Aid stations (AS) were located at approximately 25 km intervals, excluding CP sections, and CPs were positioned every 50 km. Both AS and CPs provided carbohydrate-based snacks and beverages. Split times were verified only at CPs, and runners who failed to pass within the time limit were disqualified. Among the 23 participants in this race, 18 provided complete blood samples and were included in the final analysis. At the start of the race, ambient temperature and relative humidity were 22 °C and 40%, respectively.

For the 622 km event, eligibility was restricted to runners who had completed an ultramarathon of at least 200 km within the preceding 2 years. The course extended from Ttangkkeut Village in Haenam, Jeollanam-do, to Goseong, Gangwon-do, with a cut-off time of 150 h. CPs were established at 50 km intervals to record split times and provide carbohydrate-based meals and beverages, and runners who failed to meet the cut-off time were disqualified. From the 400 km mark onward, additional AS were positioned every 25 km, without overlapping with CPs, to provide meals and beverages. Of the 28 runners who started the race, 9 completed the blood sampling protocol and were included in the final analysis. At the start of the race, ambient temperature and relative humidity were 25 °C and 50%, respectively.

Two weeks before each event, all participants underwent a cardiopulmonary exercise test (CPET) to assess baseline cardiorespiratory fitness, including resting and exercise heart rate, blood pressure, and maximal oxygen uptake (VO_2_max). A total of 44 runners were included in the final analyses. Venous blood samples were obtained from the antecubital vein within 2 h before race start and generally within 5 min after race completion to assess endocrine hormones, insulin-related markers, and lipid profiles. Runners who withdrew, failed to finish within the cut-off time, or did not provide post-race blood samples were excluded from the final analysis. The study protocol was approved by the Institutional Review Board of the authors’ institution and was conducted in accordance with the Declaration of Helsinki (IRB No. SSWUIRB-2025-028).

### 2.2. Graded Exercise Test

Graded exercise testing (GXT) was performed on a treadmill (Medtrack ST 55, Quinton Instrument Co., Boston, MA, USA) using the standard Bruce protocol. Expired gases were analyzed with a Quinton metabolic cart (QMC; Quinton Instrument Co., Boston, MA, USA) in mixing-chamber mode, and oxygen uptake (VO_2_) was recorded as 15 s averaged values. A 12-lead electrocardiogram was continuously monitored throughout the test using a Quinton stress test system (Q4500, Quinton Instrument Co., Boston, MA, USA). Resting blood pressure was measured twice at 3 min intervals after 5 min of seated rest using an automated sphygmomanometer (Model 412, Quinton Instrument Co., Boston, MA, USA), and the lower value was retained for analysis. During exercise, blood pressure was measured at the 2 min point of each stage using the same device. To improve measurement accuracy, a microphone was placed beneath the cuff to directly auscultate Korotkoff sounds for determination of systolic and diastolic blood pressure. The test followed a symptom-limited incremental protocol, and termination criteria were based on the ACC/AHA guidelines [19]. After test termination, participants walked at 2.7 km/h for 5 min during recovery while hemodynamic parameters were continuously monitored.

### 2.3. Blood Sampling and Biochemical Analysis

Venous blood samples were obtained from the antecubital vein within 2 h before race start and generally within 5 min after race completion. Pre-race blood sampling was conducted during the 2 h period preceding each event. Samples were collected during the 15:00–17:00 pre-race window for the 100 and 308 km events, both of which started at 17:00, and during the 04:00–06:00 pre-race window for the 622 km event, which started at 06:00. Pre-race samples were obtained under non-fasting conditions after participants had consumed food provided by the race organizers. During the races, nutritional intake was not standardized; participants consumed foods and beverages provided at checkpoints or aid stations and could additionally consume self-selected foods, energy gels, or bars. The interval between the last food intake and post-race blood sampling was not systematically recorded.

After race completion, participants were escorted directly from the finish area to the adjacent blood sampling area, without a planned recovery interval before venipuncture. Although the exact finish-to-venipuncture interval was not prospectively recorded, blood collection was generally initiated within 5 min of race completion. Official individual completion times were therefore used as a close proxy to characterize the approximate post-race sampling time distribution, with median and range values presented in Supplementary Table S2.

Samples were collected in serum separation tubes, centrifuged at 1509× *g* for 10 min to isolate serum, and stored at −80 °C until analysis.

Endocrine biomarkers included luteinizing hormone (LH), follicle-stimulating hormone (FSH), thyroid-stimulating hormone (TSH), triiodothyronine (T3), free thyroxine (FT4), insulin, and C-peptide. Lipid parameters included total cholesterol (TC), low-density lipoprotein cholesterol (LDL-C), high-density lipoprotein cholesterol (HDL-C), and triglycerides (TGs).

LH, FSH, TSH, T3, and FT4 were quantified using chemiluminescent immunoassays on a Cobas e602 analyzer (Roche Diagnostics GmbH, Mannheim, Germany), whereas lipid profiles were measured using enzymatic colorimetric methods on a Cobas 8000 modular system (Roche Diagnostics GmbH, Mannheim, Germany). LDL-C was measured directly using an enzymatic colorimetric method on the Cobas 8000 modular system (Roche Diagnostics GmbH, Mannheim, Germany); the Friedewald equation was not used. Insulin and C-peptide were measured by immunoassay using an Architect i1000SR analyzer (Abbott Laboratories, Abbott Park, IL, USA). For each participant, paired pre- and post-race samples were analyzed within the same assay batch to minimize inter-assay variability. All assays were performed according to the manufacturers’ standardized protocols, and analytical precision was maintained with a coefficient of variation of <5%. Hemoglobin and hematocrit were analyzed using a Beckman Coulter LH 750 (Beckman Coulter, Inc., Brea, CA, USA). Changes in plasma volume (ΔPV) were calculated separately for each participant from pre- and post-race hemoglobin (Hb) and hematocrit (Hct) values using the Dill–Costill equation [20]:

ΔPV (%) = 100 × [(Hb_pre/Hb_post) × {(100 − Hct_post)/(100 − Hct_pre)} − 1].

Post-race serum biomarker concentrations were then corrected for participant-specific plasma-volume shifts using the following equation:

C_post,corrected = C_post,measured × (1 + ΔPV/100),

where C_post,measured represents the measured post-race serum concentration and C_post,corrected represents the corresponding plasma-volume-corrected concentration. Pre-race values were used as the reference values and were not adjusted. Both measured (uncorrected) and plasma-volume-corrected biomarker concentrations are provided in Supplementary Table S1.

### 2.4. Statistical Analysis

All statistical analyses were performed using SPSS Statistics (version 26; IBM Corp., Armonk, NY, USA). One-way ANOVA was used to compare baseline characteristics among the 100, 308, and 622 km groups. Data normality was assessed using the Shapiro–Wilk test, and homogeneity of variance was examined using Levene’s test. When parametric assumptions were satisfied, Tukey’s honestly significant difference (HSD) test was used for post hoc comparisons; otherwise, the Kruskal–Wallis test with Dunn–Bonferroni post hoc analysis was applied.

Changes in endocrine and metabolic biomarkers were examined using a two-way repeated-measures ANOVA with time (pre- vs. post-race) as the within-subject factor and event group (100, 308, and 622 km) as the between-subject factor. When a significant time × event group interaction or a significant main effect was identified, post hoc simple-effect analyses were performed to determine the source of the difference.

Within-group comparisons were conducted using paired *t*-tests when normality assumptions were met and Wilcoxon signed-rank tests when distributions were non-normal. Between-group differences were examined using one-way ANOVA followed by Tukey’s HSD test or, where appropriate, the Kruskal–Wallis test with Dunn–Bonferroni correction. Effect sizes for repeated-measures ANOVA were expressed as partial eta-squared (η^2^p) to aid interpretation of the magnitude of observed effects. Because the 622 km group included a relatively small number of finishers, effect sizes were interpreted alongside *p* values when evaluating biologically meaningful findings. Data are presented as mean ± standard deviation (SD) or median (interquartile range), as appropriate, and statistical significance was set at *p* < 0.05 (two-tailed).

## 3. Results

### 3.1. Participant Characteristics

Participant characteristics across the three ultramarathon events are presented in Table 1. No significant differences were observed among the three groups (100 km, 308 km, and 622 km groups) in baseline cardiorespiratory fitness indicators, including age, anthropometric measurements, resting and maximal hemodynamic variables, and maximal oxygen consumption (VO_2_max). Regarding training characteristics, there were no intergroup differences in exercise frequency, exercise history, perceived training intensity, or the number of completed marathons. Perceived training intensity was assessed using the Borg RPE scale and reflects habitual training intensity rather than perceived exertion during the ultramarathon events. However, average daily exercise time was significantly lower in the 308 km and 622 km groups than in the 100 km group (*p* < 0.05).

Self-reported personal-best marathon completion time was significantly longer in the 622 km group than in the 100 km and 308 km groups, whereas ultramarathon completion time and mean event speed based on total elapsed time differed significantly among event groups, reflecting differences in event duration and pacing characteristics (*p* < 0.05). Mean event speed was calculated as race distance divided by total elapsed completion time and therefore includes rest and sleep periods; it should not be interpreted as moving speed or physiological exercise intensity.

### 3.2. Hematological Profiles and Plasma Volume Changes

Table 2 summarizes the hematological profiles and participant-level changes in plasma volume (ΔPV) across the three ultramarathon events. Hemoglobin increased significantly from pre- to post-race in the 100 km group (14.15 ± 0.69 to 14.55 ± 0.76 g/dL, *p* = 0.006), whereas it decreased significantly in the 308 km group (14.18 ± 1.49 to 12.99 ± 1.30 g/dL, *p* < 0.001) and the 622 km group (14.16 ± 1.27 to 12.63 ± 0.86 g/dL, *p* = 0.003). Hematocrit did not change significantly in the 100 km group (41.92 ± 2.03 to 42.25 ± 2.23%, *p* = 0.422) but decreased significantly in the 308 km group (41.42 ± 3.88 to 38.30 ± 3.39%, *p* < 0.001) and 622 km group (40.26 ± 3.29 to 36.88 ± 2.66%, *p* = 0.009).

Plasma volume change was recalculated individually for each participant using the Dill–Costill equation. The 100 km group showed a mean plasma volume contraction of −3.12 ± 6.10%, whereas the 308 and 622 km groups showed plasma volume expansions of 15.87 ± 14.63% and 19.09 ± 14.96%, respectively. ΔPV differed significantly among the event groups (Kruskal–Wallis H = 20.27, *p* < 0.001). Dunn–Bonferroni post hoc comparisons showed significantly greater plasma volume expansion in both the 308 and 622 km groups than in the 100 km group (both adjusted *p* < 0.001), whereas no significant difference was observed between the 308 and 622 km groups.

### 3.3. Changes in Endocrine and Insulin-Related Markers

Changes in endocrine and insulin-related markers after participant-level correction for plasma-volume changes are presented in Table 3.

LH showed a significant time × event group interaction (*p* = 0.049, η^2^p = 0.136). LH decreased significantly in the 100 km group, whereas no significant within-group changes were observed in the 308 or 622 km groups. At the post-race measurement, LH concentrations were significantly higher in both the 308 and 622 km groups than in the 100 km group.

FSH showed a significant main effect of time (*p* < 0.001, η^2^p = 0.340), without significant group or interaction effects. FSH decreased significantly in the 100 and 308 km groups, whereas the within-group change in the 622 km group was not significant. TSH showed no significant time, group, or interaction effects.

T3 and FT4 both showed significant main effects of time (both *p* < 0.001), without significant time × event group interactions. T3 increased significantly across all three event groups. FT4 increased significantly in the 100 and 308 km groups after adjustment for multiple within-group comparisons.

C-peptide showed a significant main effect of time (*p* < 0.001, η^2^p = 0.557) and a significant time × event group interaction (*p* = 0.008, η^2^p = 0.209). C-peptide decreased significantly in the 100 and 308 km groups, whereas the change in the 622 km group was not significant. Post-race C-peptide concentrations were significantly higher in the 622 km group than in both the 100 and 308 km groups.

Insulin showed a significant main effect of time (*p* < 0.001, η^2^p = 0.563), without significant group or time × event group effects. Insulin decreased significantly from pre- to post-race in all three event groups.

### 3.4. Changes in Lipid Profiles

**Figure 2:**
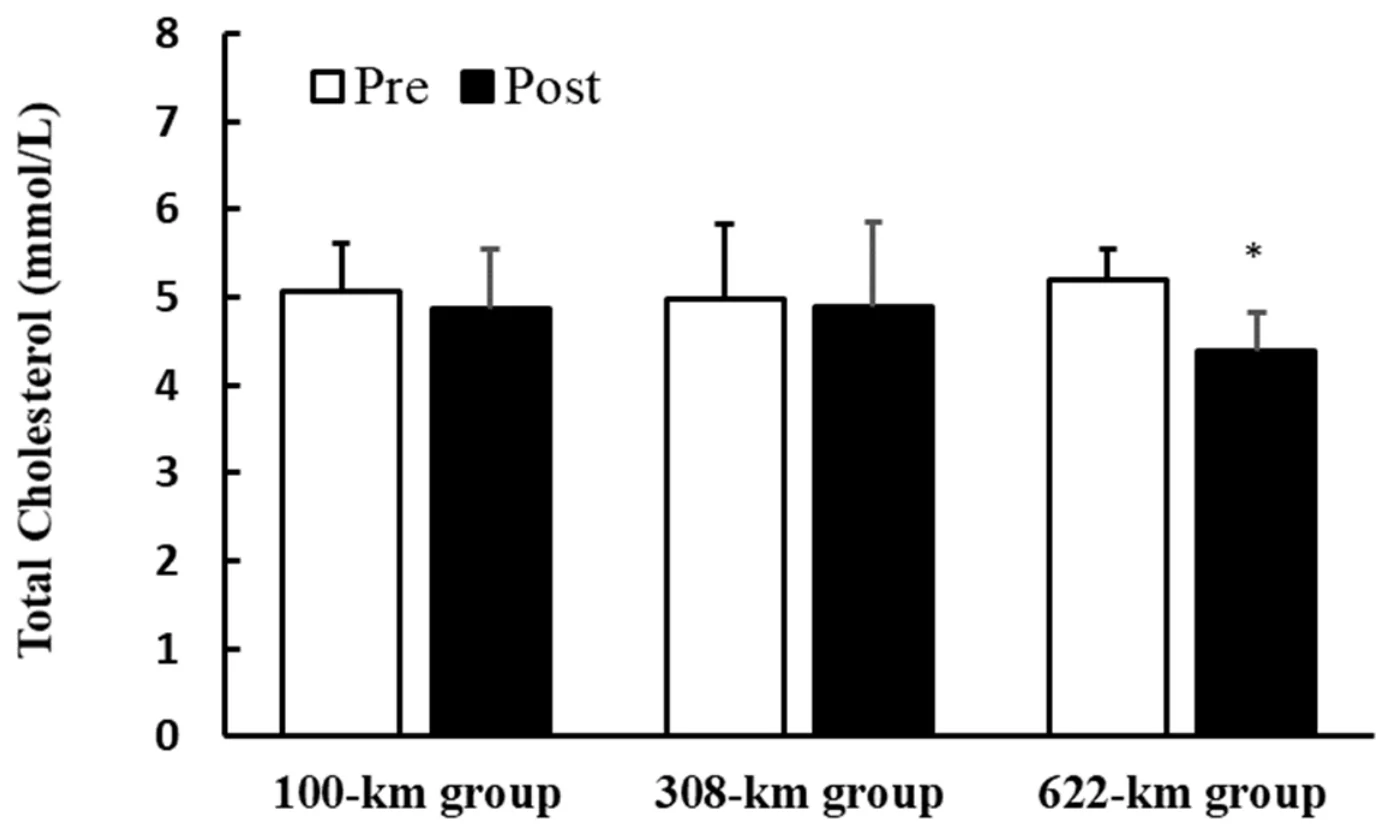
Changes in total cholesterol levels before and after ultramarathon races by event group. Data are presented as mean ± SD (100 km group, *n* = 17; 308 km group, *n* = 18; 622 km group, *n* = 9). *: Significant difference between pre- and post-values (*p* < 0.05). Time: *p* < 0.001 (η^2^p = 0.439); group: *p* = 0.799 (η^2^p = 0.011); time × event group interaction: *p* < 0.001, η^2^p = 0.328.

**Figure 3:**
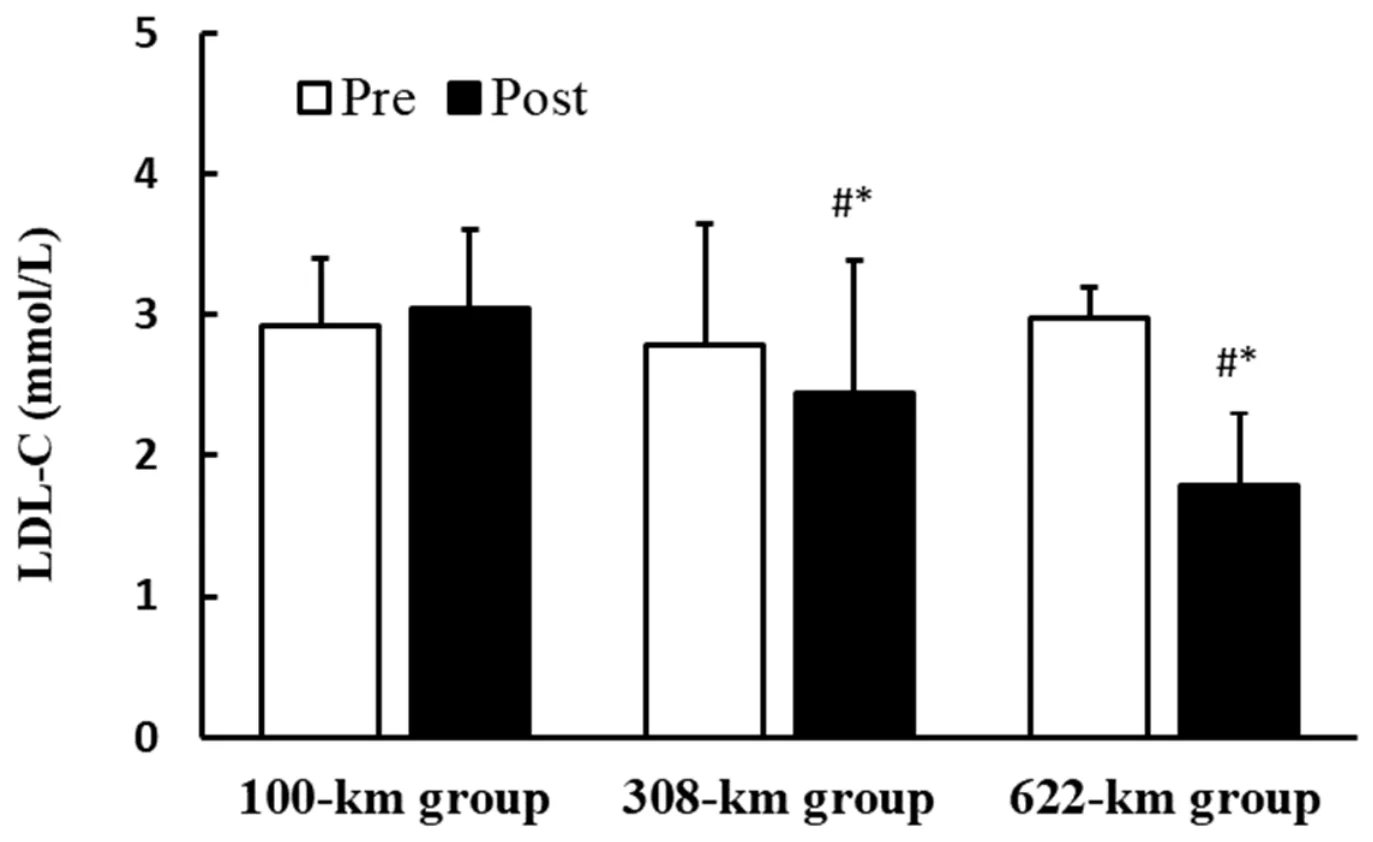
Changes in LDL-C levels before and after ultramarathon races by event group. Data are presented as mean ± SD (100 km group, *n* = 17; 308 km group, *n* = 18; 622 km group, *n* = 9). LDL-C: low-density lipoprotein cholesterol. *: Significant difference between pre- and post-values (*p* < 0.05); #: significant difference compared with the 100 km group at post-test (*p* < 0.05). Time: *p* < 0.001 (η^2^p = 0.494); group: *p* = 0.064 (η^2^p = 0.125); time × event group interaction: *p* < 0.001, η^2^p = 0.528.

**Figure 4:**
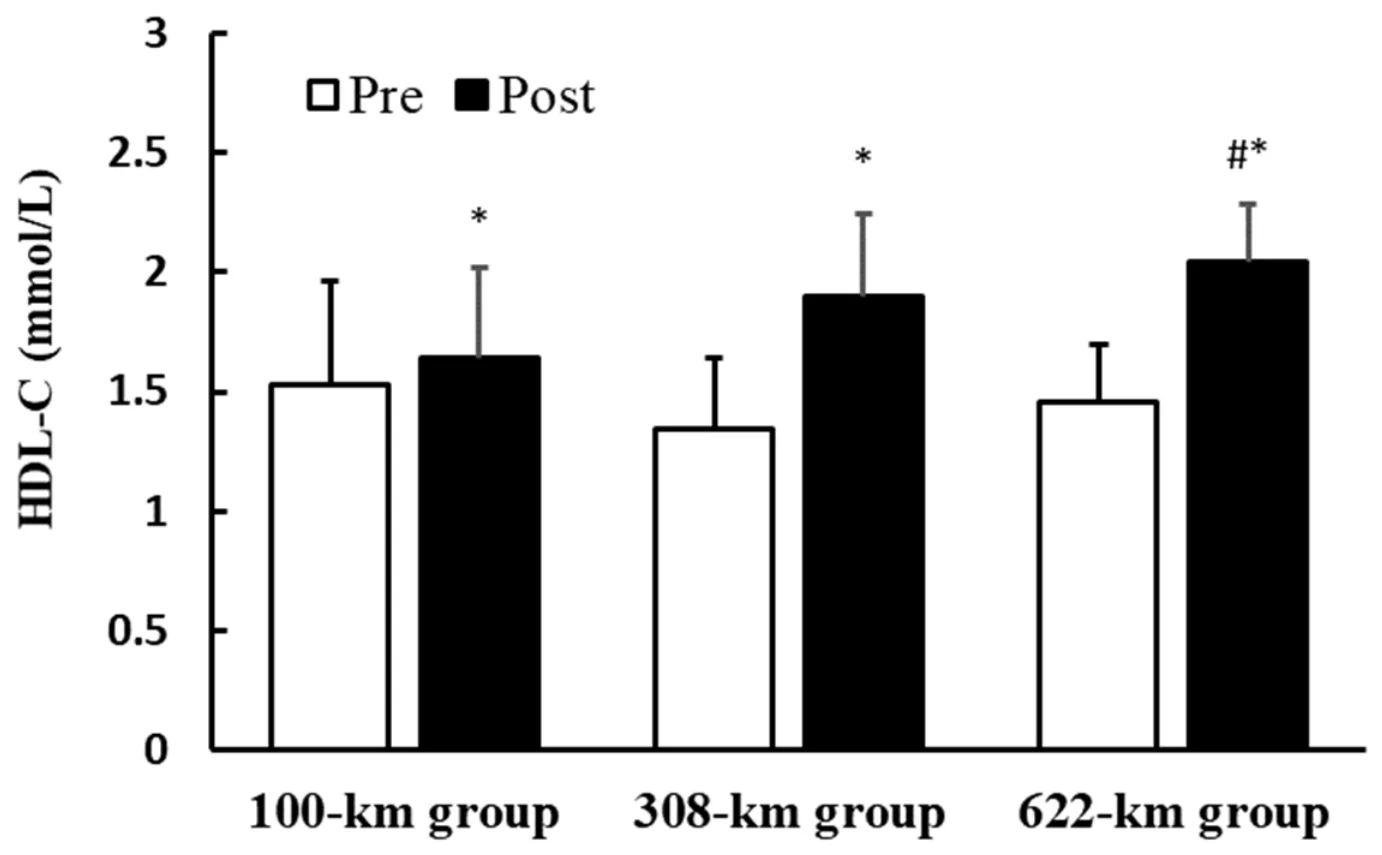
Changes in HDL-C levels before and after ultramarathon races by event group. Data are presented as mean ± SD (100 km group, *n* = 17; 308 km group, *n* = 18; 622 km group, *n* = 9). HDL-C: high-density lipoprotein cholesterol. *: Significant difference between pre- and post-values (*p* < 0.05); #: significant difference compared with the 100 km group at post-test (*p* < 0.05). Time: *p* < 0.001 (η^2^p = 0.804); group: *p* = 0.475 (η^2^p = 0.036); time × event group interaction: *p* < 0.001, η^2^p = 0.570.

**Figure 5:**
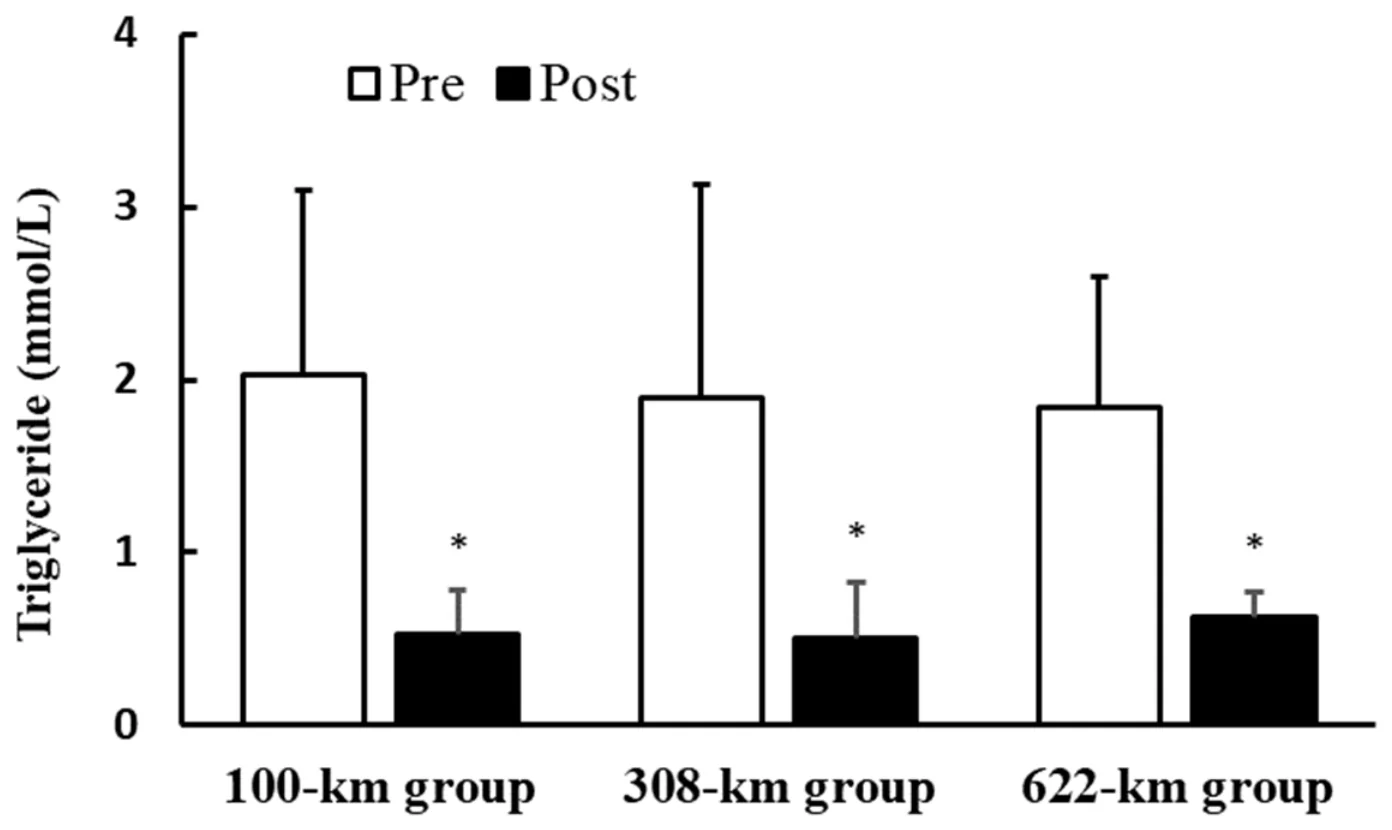
Changes in triglyceride levels before and after ultramarathon races by event group. Data are presented as mean ± SD (100 km group, *n* = 17; 308 km group, *n* = 18; 622 km group, *n* = 9). *: Significant difference between pre- and post-values (*p* < 0.05). Time: *p* < 0.001 (η^2^p = 0.621); group: *p* = 0.927 (η^2^p = 0.004); time × event group interaction: *p* = 0.812, η^2^p = 0.010.

Plasma-volume-corrected lipid responses are presented in Figure 2, Figure 3, Figure 4 and Figure 5. After participant-level correction for plasma volume changes, significant time × event group interactions remained for TC (*p* < 0.001, η^2^p = 0.328), LDL-C (*p* < 0.001, η^2^p = 0.528), and HDL-C (*p* < 0.001, η^2^p = 0.570).

For TC (Figure 2), the previously apparent reduction in the 308 km group was substantially attenuated after plasma-volume correction and was no longer significant. A significant reduction remained in the 622 km group. LDL-C (Figure 3) decreased significantly in the 308 and 622 km groups but not in the 100 km group. At post-race, LDL-C was significantly lower in both the 308 and 622 km groups than in the 100 km group.

HDL-C (Figure 4) increased significantly after the race in all three groups. Post-race HDL-C was significantly higher in the 622 km group than in the 100 km group. TGs (Figure 5) decreased significantly in all three event groups, with a significant main effect of time (*p* < 0.001, η^2^p = 0.621) but no significant time × event group interaction (*p* = 0.812, η^2^p = 0.010).

## 4. Discussion

The present study compared acute endocrine and lipid responses before and after 100, 308, and 622 km ultramarathon events. The most consistent metabolic finding was a clear insulin-related response: insulin decreased significantly across all three events, accompanied by significant C-peptide reductions in the 100 and 308 km groups. TGs also decreased and T3 and HDL-C increased across all three events, whereas FSH decreased and FT4 increased significantly in the 100 and 308 km groups. Beyond these shared responses, LH, TC, and LDL-C showed event-associated patterns, indicating that endocrine and lipid responses were not uniformly amplified with increasing race distance but differed across event contexts. These findings support extreme ultramarathon running as a useful human model for examining acute endocrine and metabolic responses under prolonged endurance stress.

The event-associated differences in circulating LH should be interpreted cautiously in light of the pulsatile nature of LH secretion in men [5]. Because only a single venous sample was obtained at each pre- and post-race time point, the measured LH concentrations may have reflected different phases of the underlying secretory pulse rather than stable changes in gonadotropin regulation [21]. Accordingly, the observed LH pattern is best regarded as a single-time-point concentration response and does not permit conclusions regarding LH pulse dynamics or hypothalamic–pituitary–gonadal axis suppression. Previous research has reported marked reductions in LH, FSH, and testosterone after a 161 km ultramarathon, supporting the concept that prolonged endurance exercise can transiently alter reproductive endocrine regulation in men [6]. Exercise-related hypogonadal responses have also been discussed as either dysfunction or adaptive regulatory adjustment in exercising males [7]. In the present study, FSH decreased significantly in the 100 and 308 km groups, whereas LH showed a more divergent event-associated response. The significant reduction in LH after the 100 km event represents an acute event-associated response. Because physiological race intensity was not directly measured, this finding cannot be attributed to greater relative exercise intensity. Differences among events in elapsed duration, pacing structure, nutritional exposure, recovery opportunities, and participant characteristics may have contributed to the observed pattern, but their individual effects cannot be separated in the present observational design. Moreover, the 100 km group reported longer daily training time, and VO_2_max was numerically higher, although the between-group difference in VO_2_max was not statistically significant; therefore, event-level characteristics cannot be fully separated from cohort-level differences. This response may reflect the combined influence of rapid energy depletion, sympathetic activation, and stress-hormone responses during prolonged endurance exercise [7,22,23].

In contrast, no significant within-group change in LH was observed in the 622 km group. A similar pattern was reported after a 622 km ultramarathon, in which hormonal responses differed according to race distance and recovery period [24]. This divergent response may reflect the distinct physiological context of multi-day ultra-endurance exercise. Compared with the 100 km group event, the 622 km event had a substantially longer elapsed duration and a lower mean event speed calculated from total elapsed time, together with repeated opportunities for food intake and brief rest. These event-level characteristics provide context for the divergent LH pattern; however, they do not establish differences in physiological exercise intensity between the events. These event characteristics may have contributed to the different post-race hormonal pattern observed in the 622 km event, although the underlying mechanisms cannot be determined from the present data. Therefore, the LH and FSH findings should be interpreted as acute changes in circulating gonadotropin concentrations after ultramarathon running rather than as evidence of altered gonadotropin regulation.

Thyroid hormone responses showed an acute post-race pattern across events. T3 increased significantly in all three event groups, whereas FT4 increased significantly in the 100 and 308 km groups and TSH remained unchanged. Previous studies have reported transient alterations in thyroid and stress-related hormones after ultra-endurance running, including changes after Spartathlon and other ultramarathon events [25,26,27]. The observed thyroid-hormone changes without a parallel TSH response may reflect peripheral redistribution, altered binding dynamics, or changes in hormone clearance rather than a purely TSH-driven stimulation of thyroid secretion [8,28]. These responses are biologically plausible because thyroid hormones regulate basal metabolic rate, mitochondrial function, oxygen consumption, and lipid metabolism during prolonged energy demand [8,9].

However, interpretation of the present thyroid findings is limited by differences in sampling time. Pre-race sampling was conducted in the afternoon for the 100 and 308 km events but in the early morning for the 622 km event, while post-race sampling times varied according to individual finishing times. Because TSH exhibits marked circadian variation, with a nocturnal surge and a daytime nadir [29], time-of-day effects may have contributed to the observed thyroid profile. Accordingly, the independent contributions of ultramarathon exercise and diurnal variation cannot be separated in the present field study. Thus, the thyroid hormone findings should be interpreted as acute event-associated responses rather than as evidence of thyroid dysfunction.

Insulin decreased significantly across all three events, whereas C-peptide decreased significantly in the 100 and 308 km groups but not in the 622 km group. These findings are consistent with event-associated changes in pancreatic β-cell secretion and substrate regulation during prolonged endurance exercise [10,11,30]. C-peptide is a useful marker of endogenous insulin secretion because it is co-secreted with insulin and is not subject to hepatic first-pass extraction [31].

However, because pre-race samples were non-fasting and the timing of the last food intake was not recorded, feeding status may have contributed to these responses. Nevertheless, the consistent decrease in insulin across all three events represents an important event-associated metabolic finding. The higher post-race C-peptide concentration in the 622 km group may reflect the distinct feeding and recovery context of this multi-day event, although this interpretation remains tentative.

The lipid responses observed in this study further illustrate event-associated differences in post-race metabolic profiles. HDL-C increased and TGs decreased after all three events, whereas TC decreased significantly only after the 622 km event and LDL-C decreased after the 308 and 622 km events. Prolonged endurance exercise increases reliance on fatty acid mobilization and oxidation, particularly as carbohydrate availability becomes limited [14,32]. Endurance exercise is also known to modify plasma lipid and lipoprotein metabolism [33].

Because pre-race samples were non-fasting and nutritional intake during the races was not standardized, feeding status may have contributed to the TG reduction, which is therefore best regarded as an event-associated post-race response rather than an effect attributable solely to exercise.

The decreases in LDL-C after the 308 and 622 km groups events and in TC after the 622 km event event further indicate that individual lipid fractions did not respond uniformly across event contexts.

However, the acute lipid profile observed after ultramarathon running should not be interpreted as direct evidence of sustained cardiometabolic benefit. TC and LDL-C reductions immediately after extreme endurance exercise may reflect transient substrate utilization, hepatic lipoprotein handling, inflammation-related lipid redistribution, and recovery-related metabolic shifts [16,17,18]. In particular, severe systemic inflammation after ultra-endurance running has been associated with acute changes in lipid and lipoprotein profiles [17]. Because inflammatory and muscle-damage markers were not measured, the contribution of inflammation-related lipid redistribution to these acute lipid changes cannot be established.

The present findings highlight the importance of viewing the 100, 308, and 622 km ultramarathon events as distinct physiological stress models rather than as a simple linear distance gradient. The 100 km event was shorter and had a higher mean event speed based on total elapsed time, whereas the 622 km event represented an extreme multi-day exposure characterized by prolonged cumulative duration, repeated nutritional intake, and intermittent rest. These measured and descriptive features distinguish the event contexts; however, they should not be interpreted as evidence that the 100 km event imposed greater physiological exercise intensity. Accordingly, the observed endocrine and lipid patterns are more appropriately interpreted as event-associated responses rather than intensity-dependent effects. Overall, the endocrine and lipid responses were event-specific rather than uniformly graded by race distance. Although some cholesterol fractions showed significant changes only in the longer events, the observed patterns cannot be attributed to any single event characteristic. Thus, endocrine and lipid responses to ultramarathon running may vary according to the broader event context, including cumulative duration, pacing characteristics, nutritional exposure, hydration status, sleep deprivation, environmental conditions, and recovery opportunities.

Several limitations should be acknowledged. First, the sample size was relatively small, particularly in the 622 km group, which may have limited statistical power and the stability of some interaction effects. Second, LH was assessed from a single venous sample at each pre- and post-race time point. Because LH secretion is pulsatile, these measurements cannot characterize pulse frequency, pulse amplitude, or overall gonadotropin regulation, and pulse-phase variability cannot be excluded. Future studies should use serial, closely spaced sampling before and after ultramarathon events to distinguish true regulatory changes from pulse-phase variability. Third, sampling time and feeding status were not standardized. Pre-race samples were obtained under non-fasting conditions, nutritional intake during the races varied, and the interval between the last food intake and post-race sampling was not recorded. Because post-race sampling times varied with individual completion times, feeding- and time-of-day-related effects cannot be separated from the observed event-associated responses. Hydration, caffeine or supplement use, sleep, and rest periods were also not standardized or individually recorded. Fourth, several biomarkers relevant to mechanistic and safety interpretation were unavailable. Testosterone, cortisol, and sex hormone-binding globulin were not measured, precluding assessment of HPG-axis suppression; renal function and electrolyte markers were not assessed, limiting interpretation of renal stress and medical safety; and markers of fat mobilization, muscle damage, and inflammation were unavailable, limiting mechanistic interpretation of the lipid findings. Fifth, only pre- and immediate post-race responses were assessed, so recovery trajectories and the persistence of endocrine or metabolic changes could not be determined; serial recovery sampling will be needed in future studies. Sixth, physiological race intensity was not directly quantified using heart rate, GPS-derived moving speed, race-specific RPE, blood lactate, or percentage of maximal oxygen uptake. Mean event speed was based on total elapsed time and therefore included rest and sleep periods, particularly in the multi-day events, and should not be interpreted as physiological exercise intensity. Future studies should incorporate continuous physiological and movement-based monitoring. Finally, the study included only experienced middle-aged male runners, and environmental conditions differed among events, limiting generalizability to other populations and race settings.

## 5. Conclusions

The most consistent metabolic finding across the three ultramarathon events was a significant post-race decrease in insulin, accompanied by significant C-peptide reductions in the 100 and 308 km groups. Among the gonadotropins, FSH decreased significantly in the 100 and 308 km groups, whereas LH decreased significantly only in the 100 km group. However, because LH was assessed from a single venous sample at each pre- and post-race time point, this finding should be regarded as a change in circulating concentration rather than evidence of altered gonadotropin regulation. Thyroid hormones showed an acute post-race pattern, with T3 increasing in all three groups, FT4 increasing in the 100 and 308 km groups, and TSH remaining unchanged. Lipid responses were partly shared and partly event-specific: TC decreased only after the 622 km event, LDL-C decreased after the 308 and 622 km events, HDL-C increased across all three events, and TGs decreased across all three events. Overall, these findings indicate a consistent insulin-related metabolic response alongside distinct gonadotropin, thyroid, and lipid response profiles across extreme ultramarathon event contexts, rather than a uniform progressive response with increasing race distance.

## Figures and Tables

**Table 1:** Participant characteristics: demographics, hemodynamic measures, and cardiorespiratory fitness.

Subject Characteristics	100 km Group (*n* = 17)	308 km Group (*n* = 18)	622 km Group (*n* = 9)	*p* Values
Age (y)	51.4 ± 4.8	49.8 ± 5.9	51.4 ± 6.7	0.653
Height (cm)	168.9 ± 4.0	168.6 ± 5.1	170.5 ± 4.5	0.596
Weight (kg)	66.7 ± 5.5	68.1 ± 6.2	69.3 ± 5.2	0.529
BMI	23.3 ± 1.7	23.9 ± 1.4	23.5 ± 1.8	0.591
Exercise frequency (sessions/week)	3.3 ± 0.9	4.0 ± 1.2	3.5 ± 1.3	0.263
Exercise history (months)	114 (84–138)	120 (88.5–134.3)	130 (120–144)	0.482
Perceived training intensity (Borg’s RPE scale)	12.1 ± 1.2	12.5 ± 1.2	12.3 ± 1.0	0.656
Exercise time (min/day)	133.9 ± 39.5	103.8 ± 23.2 *	90.0 ± 21.2 *	0.002
No. of completed marathons	39 (20–50)	40 (20–54.5)	40 (40–85)	0.385
Self-reported personal-best marathon completion time (min)	207.3 ± 17.6	201.1 ± 13.8	238.4 ± 18.5 *^†^	<0.001
Ultramarathon completion time (min)	827.0 ± 89.4	3673.3 ± 246.6 *	8728.3 ± 136.7 *^†^	<0.001
Mean event speed (km·h^−1^)	7.3 ± 0.87	5.0 ± 0.43 *	4.2 ± 0.07 *^†^	<0.001
GXT				
HR_rest_ (beats·min^−1^_)_	65.5 ± 9.3	60.2 ± 9.1	64.6 ± 10.8	0.248
SBP_rest_ (mmHg)	120.2 ± 10.0	118.2 ± 13.1	121.6 ± 12.0	0.760
DBP_rest_ (mmHg)	75.6 ± 7.1	75.8 ± 8.8	77.9 ± 7.6	0.760
HR_max_ (beats·min^−1^)	171.5 ± 10.5	171.9 ± 9.3	173.7 ± 5.1	0.839
SBP_max_ (mmHg)	219.6 ± 32.4	211.8 ± 23.1	226.3 ± 19.8	0.397
DBP_max_ (mmHg)	68.7 ± 11.6	74.6 ± 11.4	68.4 ± 11.8	0.247
TET (sec)	839.6 ± 65.8	816.1 ± 70.2	779.1 ± 73.1	0.117
VO_2_max (mL·kg^−1^·min^−1^)	50.2 ± 4.2	47.2 ± 5.2	46.1 ± 6.0	0.096

Values are means ± SD. Exercise history and number of completed marathons are presented as median (interquartile range); all other continuous variables are presented as mean ± SD unless otherwise indicated. BMI: body mass index; GXT: graded exercise test; HR: heart rate; SBP: systolic blood pressure; DBP: diastolic blood pressure; TET: total exercise time; VO_2_max: maximal oxygen consumption. *: Significantly different from 100 km group (*p* < 0.05); ^†^: significantly different from the 308 km group (*p* < 0.05).

**Table 2:** Changes in Hb, Hct, and plasma volume across ultramarathon events.

Group	Pre_Hb (g/dL)	Post_Hb (g/dL)	Pre_Hct (%)	Post_Hct (%)	Plasma Volume Change (%)	Post Hoc (Dunn–Bonferroni)
100 km	14.15 ± 0.69	14.55 ± 0.76 *	41.92 ± 2.03	42.25 ± 2.23	−3.12 ± 6.10	100 km < 308 km, 622 km
308 km	14.18 ± 1.49	12.99 ± 1.30 *	41.42 ± 3.88	38.30 ± 3.39 *	15.87 ± 14.63 ^†^	
622 km	14.16 ± 1.27	12.63 ± 0.86 *	40.26 ± 3.29	36.88 ± 2.66 *	19.09 ± 14.96 ^†^	

Values are presented as mean ± SD. ΔPV was calculated separately for each participant from individual pre- and post-race Hb and Hct values using the Dill–Costill equation and then summarized by event group. Hb: hemoglobin; Hct: hematocrit. *: Significantly different from pre-race (*p* < 0.05); ^†^: significantly different from 100 km group (*p* < 0.05).

**Table 3:** Changes in endocrine and insulin-related parameters before and after ultramarathon races across different distances.

Variable	Group	Pre	Post PV-Corrected	*p*-Values (η^2^p)
LH (mIU/mL)	100 km	4.69 ± 1.65	2.48 ± 1.40 *	T: 0.121 (0.058)
	308 km	5.35 ± 2.68	4.57 ± 2.25 ^#^	G: 0.002 (0.253)
	622 km	5.93 ± 3.01	6.73 ± 3.38 ^#^	I: 0.049 (0.136)
FSH (mIU/mL)	100 km	5.83 ± 2.11	4.23 ± 1.16 *	T: <0.001 (0.340)
	308 km	6.86 ± 3.37	5.18 ± 2.30 *	G: 0.336 (0.052)
	622 km	6.47 ± 2.14	5.88 ± 2.90	I: 0.286 (0.059)
TSH (µIU/mL)	100 km	3.76 ± 4.75	4.95 ± 5.54	T: 0.165 (0.046)
	308 km	3.60 ± 4.64	5.03 ± 4.41	G: 0.508 (0.032)
	622 km	2.66 ± 1.68	2.51 ± 1.52	I: 0.561 (0.028)
T3 (nmol/L)	100 km	1.51 ± 0.22	1.70 ± 0.26 *	T: <0.001 (0.331)
	308 km	1.32 ± 0.12	1.97 ± 1.01 *	G: 0.801 (0.011)
	622 km	1.27 ± 0.28	1.78 ± 0.38 *	I: 0.104 (0.104)
FT4 (pmol/L)	100 km	15.06 ± 2.19	16.60 ± 2.70 *	T: <0.001 (0.423)
	308 km	14.80 ± 1.80	18.53 ± 4.12 *	G: 0.578 (0.026)
	622 km	14.80 ± 1.54	17.37 ± 3.36	I: 0.125 (0.096)
C-peptide (ng/mL)	100 km	3.95 ± 1.33	1.09 ± 0.47 *	T: <0.001 (0.557)
	308 km	3.24 ± 1.31	1.54 ± 0.67 *	G: 0.067 (0.124)
	622 km	3.57 ± 1.93	2.76 ± 0.78 ^#§^	I: 0.008 (0.209)
Insulin (pmol/L)	100 km	123.76 ± 72.44	30.97 ± 23.20 *	T: <0.001 (0.563)
	308 km	97.23 ± 85.08	24.72 ± 21.32 *	G: 0.184 (0.079)
	622 km	128.76 ± 40.84	56.74 ± 34.59 *	I: 0.633 (0.022)

Values are means ± SD; η^2^p: partial eta squared; LH: Luteinizing Hormone; FSH: Follicle-Stimulating Hormone; TSH: Thyroid-Stimulating Hormone; T3: Triiodothyronine; FT4: Free Thyroxine; T: time; G: group; I: interaction effect (time x event group). *: Significant difference between pre- and post-values (*p* < 0.05); ^#^: significant difference compared with the 100 km group at post-test (*p* < 0.05); ^§^: significant difference compared with the 308 km group at post-test (*p* < 0.05).

## Data Availability

The data that support the findings of this study are available from the corresponding author upon reasonable request. The data are not publicly available because they contain information that could compromise participant privacy.

## Supplementary Materials

The following supporting information can be downloaded at https://www.mdpi.com/article/10.3390/life16091450/s1: Table S1: Measured (uncorrected) and plasma-volume-corrected endocrine and lipid biomarker concentrations before and after ultramarathon events; Table S2: Race start times, pre-race blood-sampling windows, and completion-time-based estimates of post-race sampling times.

## Abbreviations

The following abbreviations are used in this manuscript:

ACC/AHA	American College of Cardiology/American Heart Association
ANOVA	Analysis of variance
AS	Aid station
BMI	Body mass index
CK	Creatine kinase
CP	Checkpoint
CPET	Cardiopulmonary exercise test
CRP	C-reactive protein
DBP	Diastolic blood pressure
FT4	Free thyroxine
FSH	Follicle-stimulating hormone
GXT	Graded exercise test
Hb	Hemoglobin
Hct	Hematocrit
HDL-C	High-density lipoprotein cholesterol
HPG	Hypothalamic–pituitary–gonadal
HR	Heart rate
HSD	Honestly significant difference
IL-6	Interleukin-6
IRB	Institutional Review Board
LDH	Lactate dehydrogenase
LDL-C	Low-density lipoprotein cholesterol
LH	Luteinizing hormone
QMC	Quinton metabolic cart
RPE	Rating of perceived exertion
SBP	Systolic blood pressure
SD	Standard deviation
SPSS	Statistical Package for the Social Sciences
T3	Triiodothyronine
TC	Total cholesterol
TET	Total exercise time
TG	Triglycerides
TSH	Thyroid-stimulating hormone
VO_2_max	Maximal oxygen uptake
η^2^p	Partial eta-squared
