# Supplementary material for: Acute Endocrine and Lipid Responses to Extreme Ultramarathon Running Across 100, 308, and 622 km Events
Source: Life (Basel). 2026 Aug 31;16(9):1450. doi: 10.3390/life16091450 (PMC13608309; doi:10.3390/life16091450)
Supplement: Supplementary file 1 [file life-16-01450-s001.zip › Supplementary Table S2.pdf]

Supplementary Table S2. Race start times, pre-race blood-sampling windows, and completion-time-based estimates of post-race sampling times

Event	Race start	Pre-race sampling window	Pre-race status	Completion time, median (range), min	Approximate post-race sampling clock time*	Last food-to-post-sampling interval
100 km	17:00	15:00–17:00	Non-fasting	**813 (648–956)**	**Day +1, 06:33 (03:48–08:56)**	Not recorded
308 km	17:00	15:00–17:00	Non-fasting	**3740 (2754–3839)**	**Day +3, 07:20 (Day +2, 14:54 to Day +3, 08:59)**	Not recorded
622 km	06:00	04:00–06:00	Non-fasting	**8758 (8500–8890)**	**Day +6, 07:58 (03:40–10:10)**	Not recorded

Pre-race samples were obtained during the specified 2-h sampling windows under non-fasting conditions after participants had consumed food. Nutritional intake during the races was not standardized, and the interval from the last food intake to post-race sampling was not recorded. After race completion, participants were escorted directly from the finish area to the adjacent blood-sampling area without a planned recovery interval, and blood collection was generally initiated within 5 min of race completion. Exact venipuncture timestamps were not separately recorded; therefore, official individual completion times were used as a proxy for the approximate post-race sampling clock times
